# An allelic variant of GAME9 determines its binding capacity with the *GAME17* promoter in the regulation of steroidal glycoalkaloid biosynthesis in tomato

**DOI:** 10.1093/jxb/eraa014

**Published:** 2020-01-14

**Authors:** Gang Yu, Changxing Li, Lei Zhang, Guangtao Zhu, Shoaib Munir, Caixue Shi, Hongyan Zhang, Guo Ai, Shenghua Gao, Yuyang Zhang, Changxian Yang, Junhong Zhang, Hanxia Li, Zhibiao Ye

**Affiliations:** 1 The Key Laboratory of Horticultural Plant Biology, Ministry of Education, Huazhong Agricultural University, Wuhan, China; 2 The CAAS-YNNU Joint Academy of Potato Sciences, Yunnan Normal University, Kunming, China; 3 The James Hutton Institute, UK

**Keywords:** *GAME9*, *GAME17*, genome-wide association study, tomato, steroidal glycoalkaloids, variation

## Abstract

Steroidal glycoalkaloids (SGAs) are cholesterol-derived molecules found in the family Solanaceae. SGA content varies among different plant species and varieties. However, the genetic mechanisms regulating SGA content remain unclear. Here, we demonstrate that genetic variation in *GLYCOALKALOID METABOLISM* 9 (*GAME9*) is responsible for the variation in SGA content in tomato (*Solanum lycopersicum*). During a sequential analysis we found a 1 bp substitution in the AP2/ERF binding domain of *GAME9*. The 1 bp substitution in *GAME9* was significantly associated with high SGA content and determined the binding capacity of GAME9 with the promoter of *GAME17*, a core SGA biosynthesis gene. The high-SGA *GAME9* allele is mainly present in *S. pimpinellifolium* and *S. lycopersicum* var*. cerasiforme* populations and encodes a protein that can bind the *GAME17* promoter. In contrast, the low-SGA *GAME9* allele is mainly present in the big-fruited varieties of *S. lycopersicum* and encodes a protein that shows weak binding to the *GAME17* promoter. Our findings provide new insight into the regulation of SGA biosynthesis and the factors that affect the accumulation of SGA in tomato.

## Teaser

An allelic variant of the transcription factor GAME9 reduces accumulation of toxic steroidal glycoalkaloids in ripe tomato fruit via altered binding to the promoter of a steroidal glycoalkaloid biosynthesis gene.

## Introduction

Steroidal glycoalkaloids (SGAs), commonly known as *Solanum* alkaloids, are secondary metabolites primarily found in the *Solanum* family (Itkin *et al.*, 2011). These small compounds are synthesized presumably from cholesterol and typically have an oligosaccharide chain attached at the C3 position of the nitrogenous steroidal alkaloid backbone (Milner *et al.*, 2011). Since the discovery of glycoalkaloids in *Solanum nigrum* by Desfososses in 1820, approximately 100 different SGAs have been identified in various tomato tissues (Mintz-Oron *et al.*, 2008; Yamanaka *et al.*, 2008; Itkin *et al.*, 2013). These numerous structurally diverse metabolites are the most common biologically active compounds in the Solanaceae family and are strongly physiologically active in mammals (Chowanski *et al.*, 2016). SGAs primarily serve as protective agents. They deter insect herbivores and help plants to resist pathogens at all levels of biological organization. SGAs are also anti-nutritional factors because they disrupt digestion and nutrient absorption in humans (James *et al.*, 2000; Hoagland, 2009; Dolan *et al.*, 2010; Jared *et al.*, 2016).

In tomato, the major toxic SGAs—α-tomatine and its precursor—accumulate in green tissues (Kozukue *et al.*, 2004). Their toxicity is due to their ability to disrupt membranes and to their acetylcholine esterase inhibitory activity (Roddick, 1990). Indeed, a dietary concentration of 1 µmol g^−1^ tomatine inhibits the growth of *Tribolium castaneum* larvae. A total SGA level that exceeds 200 mg kg^−1^ FW of edible tuber is considered unsafe for humans (Weissenberg *et al.*, 1998; Korpan *et al.*, 2004). Reducing the concentration and glycosylation of SGAs is believed to be an effective approach for decreasing the toxicity of α-tomatine during fruit ripening (Friedman, 2004; Fujiwara *et al.*, 2004; Hoagland, 2009). Levels of this anti-nutrient were reduced or eliminated by selection and breeding during crop domestication (Itkin *et al.*, 2013). A similar story has been reported in cucumber in that bitterness was eliminated during domestication (Shang *et al.*, 2014). Increasing our understanding of mechanisms that influence the genetic control of SGA biosynthesis will contribute to our fundamental knowledge and provide a foundation for genetic improvement of important traits. However, the genetic mechanisms that influence this type of toxicity remain largely unknown.

*GLYCOALAKLOID METABOLISM* 1 (*GAME1*) was the first gene reported to encode an SGA biosynthetic enzyme (Itkin *et al.*, 2011). Subsequently a pathway for SGA biosynthesis was proposed in the Solanaceae family, based on a series of *GAME* genes (Itkin *et al.*, 2013; Nakayasu *et al.*, 2017). Extensive functional characterization provides evidence that SGAs are derived from cholesterol and that cholesterol undergoes several hydroxylation, oxidation, and transamination reactions to yield unsaturated steroidal alkaloid (SA) aglycones, which are glycosylated by different UDP-glycosyltransferases to yield SGAs (Itkin *et al.*, 2013; Sonawane et al., 2016, 2018). Unfortunately, compared with the research on the structural genes, the mechanisms that regulate SGA biosynthesis are poorly understood.

Mechanisms that coordinate the transcription of structural genes are often key regulators of complex metabolic pathways. For example, the APETALA2/Ethylene Response Factor (AP2/ERF) transcription factor ORCA2 regulates terpenoid idole alkaloid biosynthesis in *Catharanthus roseus* by binding an elicitor-responsive element in the promoter of the gene encoding Strictosidine Synthase (*Str*) (Menke *et al.*, 1999; Li *et al.*, 2013). Interactions between CrWRKY1 and *ORCA3*, a homolog of the *ORCA2* gene, were reported to influence the accumulation of ajmalicine and serpentine in the roots of *C. roseus* (Suttipanta *et al.*, 2011). In *Nicotiana tabacum*, seven AP2/ERF transcription factors encoded by the *NIC2* locus can activate alkaloid-associated gene expression by binding GCC-boxes in the promoters of genes associated with nicotine biosynthesis (Hibi *et al.*, 1994; Shoji *et al.*, 2010). The expression of ERF189, a key transcription factor for the *NIC2* locus that regulates nicotine biosynthesis, is regulated by NUP1, a plasma membrane-localized permease that takes up nicotine (Kato *et al.*, 2014).

In tomato, GAME9 serves as a master transcriptional regulator of SGA biosynthesis by binding GCC box elements in the promoters of target genes (Cárdenas *et al.*, 2016; Thagun *et al.*, 2016; Nakayasu *et al.*, 2018). In this work, we used genome-wide association study coupled with metabolomics analysis (or mGWAS) and analysed genetic variation to identify a one-base nucleotide substitution associated with variation in the SGA content in a natural population of tomato. This variation was selected during the domestication of tomato. Moreover, we confirmed that this one-base nucleotide substitution interconverts the high-SGA allele to the low-SGA allele of *GAME9*. The high-SGA *GAME9* allele is mainly represented in *Solanum pimpinellifolium* (PIM) and *S. lycopersicum* var. *cerasiforme* (CER) and encodes a protein that can bind a *cis*-element in the promoter of *GAME17*—a core gene for SGA biosynthesis. In contrast, the low-SGA allele is mainly present in the big-fruited varieties of *S. lycopersicum* (BIG) and encodes a protein that shows weak binding to the *GAME17* promoter. These findings provide insight into new strategies for regulating toxic compounds and the genetic improvement of tomato. They also provide information useful for engineering reduced levels of anti-nutritional compounds in other plants.

## Materials and methods

### Tomato accessions and genome-wide association mapping

A total of 2 678 533 single nucleotide polymorphisms (SNPs; minor allele frequency >5% and missing rate <10%) for 442 accessions were used in a GWAS, which included 31 PIM accessions, 124 CER accessions, and 287 BIG accessions that were from the same populations as used previously (Zhu *et al.*, 2018). The relative quantification of the seven SGA metabolites used for mGWAS in the current work came from the previously study (Zhu *et al.*, 2018). The association analyses were performed using the compressed mixed linear model as described previously (Zhu *et al.*, 2018). The genome-wide suggestive thresholds (*P*=1/*n*, ≤2.4×10^–7^) and signiﬁcance thresholds (*P*=0.05/*n*, ≤1.2×10^–8^) of all the SNPs were defined at a uniform threshold. VCFtools was used to perform local linkage disequilibrium (LD) analysis. The 83.9–84.2 Mb regions of SNPs (*P*<2.4×10^–7^) centered on the lead SNP (ch01:84016748) were selected to calculate the *r*^2^ of LD. The physical locations of the SNPs were identified based on tomato genomic sequence version SL2.50. All tomato plants were self-pollinated. Tomato fruit was hand-harvested at physiological maturity.

### Metabolite analysis

To get more accurate phenotypic data from transgenic plants, the fruits (peel and flesh tissues without the seeds and gel) were harvested uniformly at their mature green (MG), breaker, and red ripe (RR) stages. The freeze-dried samples (100 mg) were extracted for 8 h at 4 °C with 1.0 ml 80% methanol: water (V:V) containing 0.1 mg l^−1^ lidocaine. The samples were centrifuged for 10 min at 12 000 *g*, then filtered with an organic filter with a 0.22 µm pore size (Itkin *et al.*, 2011; Zhu *et al.*, 2018). The extracts (2 µl) were analysed using a UPLC-ESI-qTOF instrument (Agilent 6520) that was equipped with C18 eclipse plus columns (100×2.1 mm i.d., 1.8 µm particle size), a solvent of ultrapure water (eluent A) and acetonitrile (eluent B) both containing 0.1% (v/v) formic acid at a flow rate of 0.3 ml ml^−1^ according to the following procedure: 5:95 v/v B:A at 0 min, 95:5 at 20 min, 95:5 at 22 min, 5:95 at 22.1 min, and 5:95 at 26 min. The column was subsequently washed for 10 min and then equilibrated before the next injection. The column temperature was 25 °C. The sample was stored at 4 °C prior to injection (Adato *et al.*, 2009; Chen *et al.*, 2013). Full time-of-flight (TOF) scans of HPLC effluents were used to detect the *m*/*z* range from 50 to 1500 Da in positive ion mode. For MS/MS, 5–80 collision energies were used. α-Tomatine and its precursor were identified by a comparison of their retention times and MS/MS fragments with standard compounds or to values found in the literature (Cataldi *et al.*, 2005; Itkin *et al.*, 2011; Iijima *et al.*, 2013). Relative quantification of the compounds was performed with Qualitative Analysis B.04.00 (Agilent).

### RNA isolation and gene expression analysis

Total RNA was isolated using the Trizol reagent (Sigma-Aldrich). cDNAs were synthesized form DNaseI-treated total RNA using a Hiscript II 1st Strand cDNA Synthesis Kit (Vazyme, China). Expression analysis of SGA-related genes in the fruits (peel and flesh tissues without the seeds and gel) of wild-type and transgenic lines was performed using three biological replicates (*n*=3), the actin gene (BT013524) was used as an internal control. Primer sequences are list in Supplementary Table S1 at *JXB* online.

### Yeast one-hybrid assay

Promoter fragments from *GAME17* were amplified from TS-303 genomic DNA with specific primers (see Supplementary Table S1) and cloned into the bait vector pAbAi (Clontech). The full-length *GAME9*^135A^ and *GAME9*^135V^ with specific primers (Supplementary Table S1) were amplified from cDNA prepared from TS-303, a CER tomato that exhibits high SGA content and AC, a BIG tomato that exhibits low SGA content, respectively. They were then fused to the GAL4 activation domain in the prey vector pGADT7. The bait plasmids were digested, linearized, integrated into the Y1HGold yeast (*Saccharomyces cerevisiae*) genome and cultured on SD/−Ura at 30 °C for 3 d. The bait-reporter strain was transformed with the prey vector. After 3 d, the positive yeast strains were picked and diluted with sterile water. These suspensions were spotted on an SD/−Leu medium that contained from 0 to 100 ng ml^−1^ aureobasidin A. The plates were incubated for 3–4 d at 30 °C. The negative (pGADT7+N-AbAi) controls were prepared as described above. The DNA–protein interactions were analysed based on the growth of the yeast strains relative to the negative controls on media with different ABA concentrations.

### Transient expression assays in tobacco leaves

The two alleles *GAME9*^135A^ and *GAME9*^135V^ were amplified from the genomic DNA of TS-303 and AC, respectively, with specific primers (see Supplementary Table S1) and fused to pGreen II 62-SK, a CaMV 35S promoter-driven effector (Hellens *et al.*, 2005). The fragments of the promoter were cloned into the pGreen II 0800-Luc vector harboring the firefly luciferase (LUC) reporter gene and expressed under the control of the CaMV 35S promoter (Hellens *et al.*, 2005). *Agrobacterium tumefaciens* (GV2260)-mediated transformation was used to transiently express both reporters and effectors in *Nicotiana benthamiana* leaves. The empty pGreen II 62-SK was used as the negative control. Three days after the transfection, both fLUC and rLUC activities were measured using the dual luciferase assay reagents (Promega, USA) and an Infinite M200 plate reader (Tecan, USA). To generate β-glucuronidase (GUS) reporter proteins for observation, the two alleles *GAME9*^135A^ and *GAME9*^135V^ were amplified from the genomic DNA of TS-303 and AC, respectively, and fused to pHellsgate8 vector, a CaMV 35S promoter-driven effector, and the fragment of the promoter was cloned into pMV2 vector (derived from the pHellsgate8 vector with a deleted CaMV 35S promoter) harboring the GUS reporter gene. The empty pHellsgate8 was used as the negative control. Three days after the transfection, both the negative control and the fusion proteins were incubated at 37 °C for 12 h in staining buffer (100 mM sodium phosphate, pH 7, 0.1% Triton X-100, 10 mM Na_2_EDTA, 0.5 mM K_3_Fe(CN)_6_, 0.5 mM K_4_Fe(CN)_6_, and 1mg ml^−1^ 5-bromo-4-chloro-3-indolyl-β-D-glucuronic acid), followed by washing with 70% (v/v) ethanol. The relative expression of GUS reporter gene and negative control was determined with the GUS primers, and the actin gene (BT013524) was used as an internal control. Primers sequences are list in Supplementary Table S1.

### Electrophoretic mobility shift assays

The whole coding sequences of *GAME9*^135A^ and *GAME9*^135V^ were amplified from TS-303 and AC, respectively, and then inserted into pET15d. The recombinant fusion protein with a MBP-tag was expressed in *Escherichia coli* DE3 (BL21) cells (Invitrogen, USA) and affinity purified using magnetic agarose (Biolabs, USA) according to the manufacturer’s instructions. To label the probes, FAM was attached to the sense oligonucleotides at their 5′ end. Sense, antisense, and mutated oligonucleotides (see Supplementary Table S1) were annealed by gradual cooling after heating at 95 °C for 5 min. An Electrophoretic mobility shift assay (EMSA) was performed with the LightShift Chemiluminescent EMSA Kit (Thermo Fisher Scientific, USA). GAME9 protein and the probes were incubated at room temperature in 1× Binding Buffer (100 mm MgCl_2_, 2.5% (v/v) glycerol, 50 ng μl^−1^ poly (dI-dC)). After 30 min, the reaction products were separated by electrophoresis on 6% non-denaturing polyacrylamide gels. After migration, the FAM-labeled probe on the non-denaturing polyacrylamide gel was visualized using an Amersham Imager 600.

### Vector construction and tomato transformation

The full-length *GAME9* open reading frame (ORF) was amplified from TS-303. The *GAME9*-silenced fragment was amplified from AC cDNA using gene-specific primers with 5′-attB1 and 3′-attB2 extensions on the forward and reverse primers. The full-length *GAME9* ORF was cloned into overexpression vector pMV2 (derived from the pHellsgate8 vector) using a CaMV 35S promoter. The *GAME9*-silenced fragment was recombined into the RNAi vector pHellsgate 2 using the Clonase BP reaction (Invitrogen). All vectors were introduced into the *A. tumefaciens* strain C58 for tomato transformation. TS-286 was transformed with the overexpression vector driven by a CaMV 35S promoter and the silencing vector. The genomic DNA from these transgenic plants was analysed with PCR-based genotyping experiments. The pertinent primers are listed in Supplementary Table S1.

### Accession numbers

Sequence data from this article can be found in the Sol Genomics Network databases with the following accession numbers: *ERF1* (Solyc01g090300), *ERF2* (Solyc01g090310), *ERF3* (Solyc01g090320), *ERF5* (Solyc01g090370), *GAME1* (Solyc07g043490), *GAME2* (Solyc07g043410), *GAME4* (Solyc12g006460), *GAME6* (Solyc07g043460), *GAME7* (Solyc07g062520), *GAME9* (Solyc01g090340), *GAME9* homolog from potato (Sotub01g029510), *GAME11* (Solyc07g043420), *GAME12* (Solyc12g006470), *GAME17* (Solyc07g043480), *GAME18* (Solyc07g043500), and *MYC2* (Solyc08g076930).

## Results

### *GAME9* is a major locus controlling SGA content in tomato

**Fig. 1.**
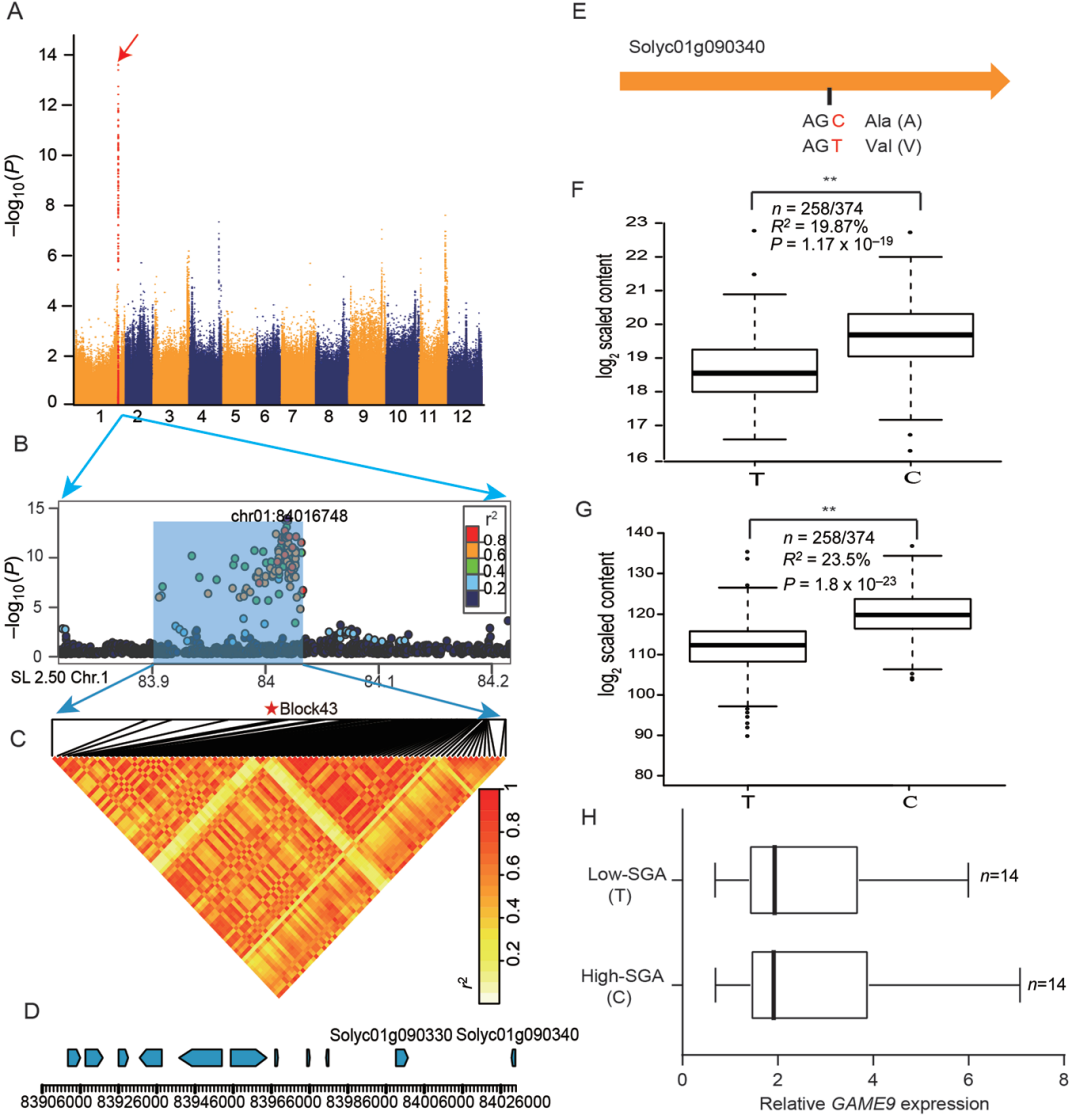
Loci associated with hydrotomatidine content. (A) Manhattan plot for a GWAS of SGA (hydrotomatidine) content. (B) Detailed plot for 83.9–84.2 Mb on chromosome 1 (*x*-axis). Lead SNP is indicated by darker shading. A representation of pairwise *r*^2^ values (a measure of LD) among all SNPs in region 83.9–84.2 Mb, where the shading of each box corresponds to the *r*^2^ value as shown by the key. (C) A representation of the pairwise *r*^2^ values among all polymorphic sites in the 122.7 kb genomic region corresponding to (B), where the shade of each box corresponds to the *r*^2^ value according to the key. Haploblock 43 (marked by star) contains lead SNP associated with fruit SGA content. (D) Gene structure of 11 genes in region 83.9–84.2 Mb, an unknown protein (*Solyc01g090330*), and an APETALA2/Ethylene Response Factor (*Solyc01g090340*) in haploblock 43. (E) Model and mutation for *GAME9*. (F) The effect of different alleles on the content of hydrotomatidine (SlFM1985). The data were plotted as a function of a particular SNP (ch01:84029382). The middle line of the box indicates the median, the box indicates the range of the 25th to 75th percentiles of the total date, the whiskers indicate the interquartile range, and the outer dots are outliers. (G) Total SGA content in different genotypes. The box plot (detailed information same as for (F)) includes measurements for hydrotomatidine (SlFM1985), hydroxytomatidenol (SlFM0960), hydroxytomatidine (SlFM0964), dehydrofilotomatine (SlFM1979), neorickiioside A (SlFM1983), lycoperoside H (SlFM1984), and lycoperoside A (SlFM1989). The data were plotted as a function of a particular SNP (ch01:84029382). (H) The relative expression of *GAME9* in red ripe tomato fruit. *n* refers to the number of appropriate genotypes of tomato accessions used in this study. (This figure is available in color at *JXB* online.)

In a previous study, using a metabolic genome-wide association, we found a SNP (ch01:84016748) that showed a strong association with the levels of hydrotomatidine (SlFM1985; *P*=2.25×10^–14^), hydroxytomatidenol (SlFM0960; *P*=8×10^–15^), hydroxytomatidine (SlFM0964; *P*=1.78×10^−10^), dehydrofilotomatine (SlFM1979; *P*=1.11×10^–7^), neorickiioside A (SlFM1983; *P*=4.45×10^–12^), lycoperoside H (SlFM1984; *P*=5.54×10^–11^) and lycoperoside A (SlFM1989; *P*=6×10^–12^) (Fig. 1; Supplementary Fig. S1) (Zhu *et al.*, 2018). Then we carefully analysed the pairwise LD distance within the 2 Mb interval centered on the SNP (ch01:84016748) from the GWAS of hydrotomatidine content (Fig. 1B). All the suggestive SNPs (*P*<2.4×10^–7^) fell into a 122.7 kb region of 83.9–84.2 Mb; there were a total of 11 genes in this region (Supplementary Table S2). A haplotype analysis of the region spanning all the significant SNPs on chromosome 1 (122.7 kb) identified 86 haploblocks (Supplementary Table S3), and many significant SNPs, including SNP ch01:84016748, can be traced back to haploblock 43 (SL2.50ch01:84014759–SL2.50ch01:84029382) (Fig. 1C). Haploblock 43 spans two genes (Fig. 1D), an unknown protein (*Solyc01g090330*), and an AP2/ERF (*Solyc01g090340*) named *GAME9*, which has been reported to regulate the biosynthesis of steroidal alkaloids (Cárdenas *et al.*, 2016). Indeed, our transgenic analysis also confirmed that the overexpression and silencing of *GAME9* in the high-SGA tomato cultivar TS-286 led to significantly increased or decreased levels of both α-tomatine and hydrotomatidine (Supplementary Fig. S2).

### A natural variant of *GAME9* associated with SGA content in tomato

To investigate functional allelic variation at the *GAME9* locus, we analysed the nucleotide sequence of *GAME9* in 374 tomato accessions with diverse SGA content. The resequencing of the *GAME9* coding region indicated that a polymorphism (SNP G/A, ch01:84029382) that causes an alanine to valine substitution at position 135 (A135V) in the N-terminus of AP2/ERF binding domain explained 19.87% and 23.5% of the variation in the level of hydrotomatidine and the seven SGAs in tomato fruit, respectively (Fig. 1E, F, G). A subsequent analysis indicated a significant difference (*P*=1.8×10^–23^, *n*=374) between the levels of the seven SGAs in the two alleles of *GAME9* (Fig. 1G). Thus, the *GAME9* genotype can be classiﬁed into two different haplotypes in tomato. We refer to the ‘low-SGA’ content phenotype as *GAME9*^135V^, which is mainly present in the BIG population (86.5%), and the ‘high-SGA’ content phenotype as *GAME9*^135A^, which is mainly present in the PIM (100%) and CER (71.2%) populations. Further, we randomly selected 14 accessions of high SGA and 14 accessions of low SGA and measured expression of *GAME9* in red ripe fruit by quantitative RT-PCR. The expression of *GAME9* showed no significant difference in red ripe fruit between high- and low-SGA accessions (Fig. 1H). These findings indicate that this allelic variant of *GAME9* may play a unique role in the transcriptional regulation of SGA.

### GAME9 specifically binds a GC-rich element in the *GAME17* promoter

Previous study showed that ERF189, ORCA3, and GAME9 can bind to a GC-rich box (5′-CCGCCCTCCA-3′) in the tobacco *PMT2* promoter (Shoji *et al.*, 2013). We discovered similar elements in the promoters of *GAME7*, *GAME4*, and *GAME17* (see Supplementary Table S4). Furthermore, expression analysis revealed that *GAME4* and *GAME17* were significantly down-regulated in the *GAME9*-RNAi lines and *GAME17* was down-regulated to a greater extent than the other *GAME* genes (Supplementary Fig. S3). Thus, we tested whether GAME9^135A^ can directly affect the transcription of *GAME17*. A yeast one hybrid (Y1H) assay indicated that GAME9^135A^ can bind to the N1 motif (−898 to −378 bp) of the *GAME17* promoter and that GAME9^135A^ cannot bind the N2 motif (−666 to −378 bp) of the *GAME17* promoter (Supplementary Fig. S4A, B). To independently test the idea that GAME9^135A^ can bind the *GAME17* promoter, we generated two promoter deletion constructs and tested whether GAME9^135A^ could induce the transcription of these genes in tobacco (*N. benthamiana*) leaves. We found that the P1 sequence (from −898 bp to the translational start codon) was essential to activate a luciferase (LUC) reporter gene. In contrast, the P2 sequence (from −671 bp to the translational start codon) was not necessary (Supplementary Fig. S4C, D). Thus, we conclude that the *GAME9*^135A^ protein can bind the core sequence between −898 and −671 bp in the *GAME17* promoter. We tested whether the GC-rich element (5′-CGCCCCCC-3′) in this core sequence could serve as a *GAME9*^135A^ binding site using an EMSA. We compared the ability of *GAME9*^135A^ to bind probes containing this GC-rich sequence with probes containing mutations in this GC-rich sequence (Supplementary Fig. S4E). We found that the *GAME9*^135A^ fusion protein can bind the wild-type *cis*-element (5′-CGCCCCCC-3′) but not the mutant *cis*-element (Supplementary Fig. S4F). These data indicate that *GAME9*^135A^ can directly bind the promoter of *GAME17* to positively activate the transcription of genes required for SGA biosynthesis.

### Allelic variation in GAME9 determines its binding capacity with *GAME17*

**Fig. 2.**
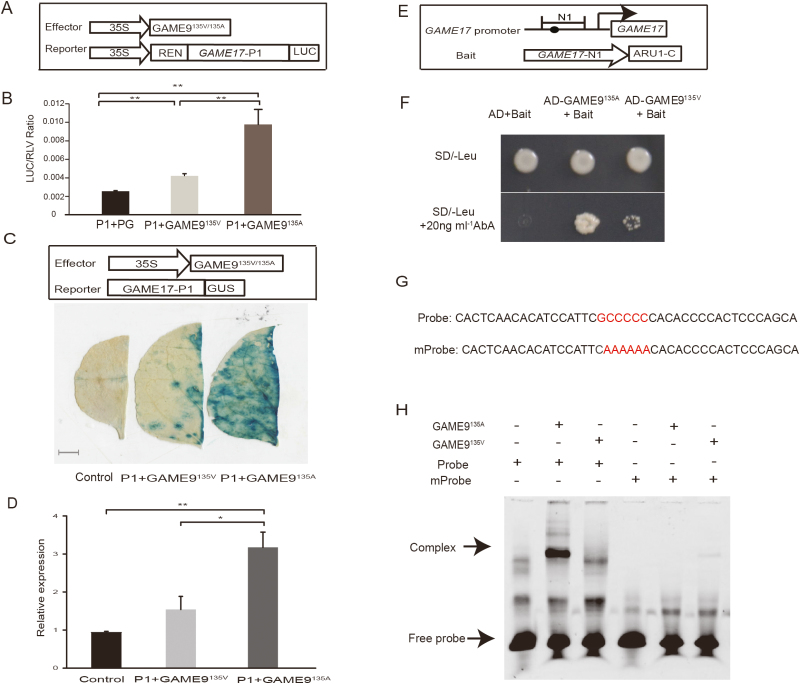
Transactivation of *GAME17* promoters by GAME9^135A^ and GAME9^135V^. (A) Schematic diagrams of the constructs used for the dual luciferase assay. The reporter constructs were created by cloning promoter fragment of GAME17, P1 (−898 bp to the translational start codon), into pGreen II 0800-Luc. The effector constructs were created by cloning the full-length open reading frames of GAME9^135A^ and GAME9^135V^ into pGreen II 62-SK. (B) Transactivation activity of GAME9^135A^ and GAME9^135V^ on the *GAME17* promoter in tobacco leaves. LUC, firefly luciferase activity; RLU, *Renilla* luciferase activity. The P1 and the pGreen II 62-SK empty (PG) vector were used as controls. Bars represent mean values ±SE (*n*>6). Asterisks indicate statistically significant differences determined using a *t*-test: **P<0.01. (C) GUS activity of GAME9135A and GAME9^135V^ on the *GAME17* promoter in tobacco leaves. Schematic diagrams of the constructs used for the GUS activity assay are shown at the top. P1: the promoter fragment of *GAME17* (−898 bp to the translational start codon). P1 and pHellsgate8 empty vector were used as controls. Scale bar, 1 cm. (D) GUS expression level driven by GAME9^135V^ and GAME9^135A^ effector. The control and fragment of GAME17 promoter correspond to those shown in (C). A validation experiment (*n*=4) was performed. Asterisks indicate statistically significant differences determined using a *t*-test: *P<0.05, **P<0.01. (E) Schematic diagrams of the *GAME17* promoter and reporter constructs used for the Y1H assay. The circles indicate the *cis*-acting GC-rich element (−724 to −717 bp) within the *GAME17* promoter. N1 (−898 to −378 bp) indicates the promoter fragments of GAME17 that were cloned into the bait plasmid pAbAi. (F) *GAME17* promoter-binding activity of GAME9^135A^ and GAME9^135V^. The promoter binding activities were determined using a Y1H assay. The bait vector and the empty pGADT7 vector were co-transformed into Y1Gold as a negative control. All transformants were grown on a selective medium containing (top) or lacking (bottom) 20 ng ml^−1^ antibiotic (AbA). (G) Wild-type and mutant probes were used for EMSAs. The wild-type probe was synthesized based on the *GAME17* promoter sequence. The *cis*-element sequence was replaced with AAAAAA in the mutant probe (mProbe). The hypothetical *cis*-element is indicated with shaded letters. (H) *In vitro* binding of GAME9^135A^ and GAME9^135V^ to the promoter of *GAME17*. −, absence; +, presence. The protein–DNA complex and free probe are indicated. (This figure is available in color at *JXB* online.)

To learn more about the influence of the SNP (ch01:84029382) that affects the N-terminal AP2/ERF domain of GAME9, we used a CaMV 35S promoter-driven *GAME9*^135V^ effector (pGreen II 62-SK) to perform transient transactivation and promoter binding assays. The infiltrated plants revealed that the P1 sequence in the *GAME17* promoter could support the transactivation activity of both *GAME9*^135V^ and *GAME9*^135A^. However, the transient overexpression of *GAME9*^135A^ could activate transcription of the luciferase reporter gene (3.8-fold) more than could the transient overexpression of *GAME9*^135V^ (1.6-fold) (Fig. 2A, B). This result was independently verified with a *GAME17* promoter-driven GUS reporter gene. The histochemical staining of GUS activity in tobacco leaves showed that with GUS expression driven by the *GAME9*^135V^ effector there was little expression relative to the control, while GUS expression driven by the *GAME9*^135A^ effector was significant relative to the control (Fig. 2C, D). Additionally, the GAME9^135V^ positive yeast strains grew more slowly and showed weak binding activity relative to the GAME9^135A^ positive yeast strains (Fig. 2E, F). Finally, EMSAs were performed to compare the binding activities of GAME9^135V^ and GAME9^135A^ to its binding site in the *GAME17* promoter *in vitro*. Although the GAME9^135A^ protein was able to retard the mobilities of *GAME17* promoter fragments containing the GC-rich element, the GAME9^135V^ protein was not able to bind the same DNA fragments (Fig. 2G, H). Taken together, *GAME9*^135A^ is a more potent activator of the *GAME17* promoter than is *GAME9*^135V^ and the protein encoded by *GAME9*^135V^—an allelic variant of *GAME9*^135A^—appears to have weak ability to bind the *GAME17* promoter.

**Fig. 3.**
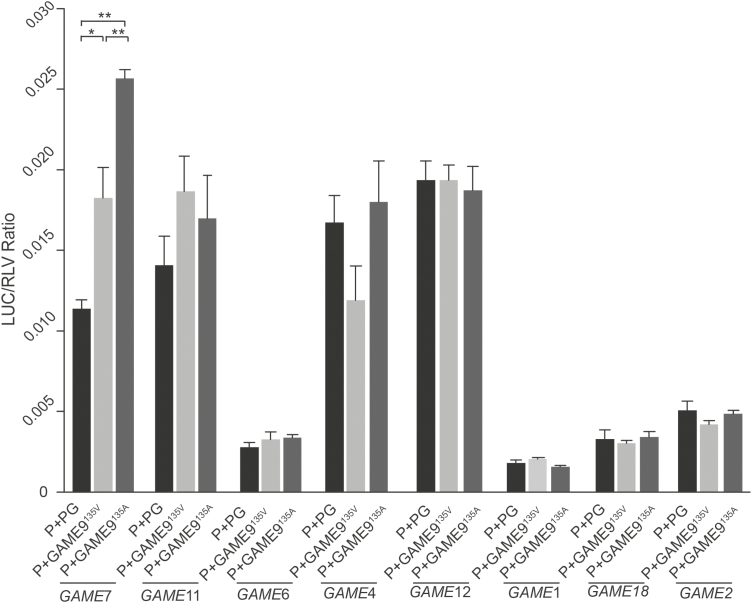
Transactivation assays of putative downstream gene promoters by GAME9^135V^ and GAME9^135A^. The capacity of GAME9^135V^ and GAME9^135A^ to transactivate eight different promoters of candidate downstream genes was evaluated in tobacco leaves. P, promoter fragment of candidate downstream genes; PG, pGreen II 62-SK empty vector. A validation experiment (*n*>8) was performed. Asterisks indicate statistically significant differences determined using a *t*-test: *P<0.05, **P<0.01.

To test whether this regulatory strategy is used for other *GAME* genes, the transactivation of *GAME7* (−682 bp to the translational start codon), *GAME11* (−1581 bp to the translational start codon), *GAME6* (−1112 bp to the translational start codon), *GAME4* (−1201 bp to the translational start codon), *GAME12* (−1612 bp to the translational start codon), *GAME1* (−1220 bp to the translational start codon), *GAME18* (−517 bp to the translational start codon), and *GAME2* (−1551 bp to the translational start codon) by *GAME9*^135A^ and *GAME9*^135V^ was tested in tobacco leaves. We found that *GAME9*^135A^ and *GAME9*^135V^ are capable of transactivating the *GAME7* promoter (Fig. 3). More importantly, a similar trend was observed with *GAME7* in that GAME9^135A^ was a more potent activator than GAME9^135V^ (Figs 2, 3). In addition, we randomly selected 14 accessions of high SGA and 14 accessions of low SGA and measured expression of *GAME17* in red ripe fruit by quantitative RT-PCR. The expression of *GAME17* showed a significant difference in red ripe fruit between high- and low-SGA accessions (see Supplementary Fig. S5). So these findings showed that *GAME9*^135A^ can bind the GC-rich element (5′-GCCNNCC-3′) in the promoters of *GAME17* and *GAME7*, and relative to GAME9^135A^, GAME9^135V^ weakly binds and activates the transcription of genes that contribute to SGA biosynthesis. Taken together, it is clear that the binding activity of GAME9 depends on allelic variation that influences the N-terminal AP2/ERF domain.

## Discussion

Alkaloids are a major and extensively studied class of plant metabolites with diverse biological activities. They are widely reported in important crop plants, such as tomato, potato (*Solanum tuberosum*), and eggplant (*Solanum melongena*) (Eich, 2008; Buckingham *et al*., 2010). Understanding the genetic mechanisms that control the accumulation of SGAs will help us to improve the nutritional quality and consumption value of important crops, but until now, most of the research on SGAs has focused on their regulation by jasmonate, the elucidation of compound structures, and SGA biosynthesis (van der Fits and Memelink, 2000; De Boer *et al.*, 2011; Itkin *et al.*, 2013; Schwahn *et al.*, 2014; Umemoto *et al.*, 2016; Sonawane *et al.*, 2018). The genetic control of SGA content—especially the core SGA biosynthesis pathway that acts between cholesterol and α-tomatine—is not entirely clear and requires further investigation. In this study, we provide multiple lines of experimental evidence that help to clarify the impact of genetic variation on regulation of SGAs in tomato.

As described above, we found that natural variation in *GAME9* had a role in regulating the accumulation of the seven SGAs during domestication. Genetically, PIM belongs to the wild tomato group that originated in the Andean region of South America and is the ancestor of the CER and BIG groups that were later dispersed to other parts of the world and subjected to selection by local farmers and breeders (Lin *et al.*, 2014). In general, because of their anti-nutritional activities, there was a strong selection against SGAs during the development of BIG from PIM (Zhu *et al.*, 2018). Changes to the GAME9 protein acquired during evolution possibly affected the accumulation of SGA metabolites because *GAME9* is a major regulator of SGA biosynthesis. Other than a distinct genetic factor, one possible reason for the variation of SGA content during domestication could lie in the base pair substitution in the coding sequence of *GAME9* differentially regulating SGA biosynthesis. Beyond that, our analysis revealed that *GAME9* is located inside a tandem duplication of an ERF gene and that among the natural populations we analysed, *GAME9* is the only gene in the tandem duplication encoding a protein with a serine-rich C-terminal domain (see Supplementary Fig. S6). This finding indicates that lack of the serine-rich domain in tomato GAME9-like proteins prevents these proteins from directly participating in SGA biosynthesis but does not exclude a possible indirect contribution to SGA biosynthesis.

Functional characterization by transient luciferase expression assays, Y1H assay and EMSA showed that GAME9 can directly regulate transcription from the *GAME7* and *GAME17* promoters (Figs 2, 3). Furthermore, a similar element in the promoters of *GAME7* and *GAME17* was discovered (see Supplementary Table S4). These finding indicated that GAME9 can directly bind a GC-rich sequence that is a highly conserved functional element (5′-GCCNNCC-3′) in the promoter regions of *GAME17* and *GAME7* rather than the canonical GCC box to regulate the core SGA biosynthetic pathway, although GAME9 has substantial GCC box-binding activity *in vitro*. More importantly, the allelic variation located in the region of *GAME9* encoding the N-terminal AP2/ERF domain has a decisive effect on the binding activity of GAME9 (Fig. 2). The GAME9-based regulatory strategy that controls SGA biosynthesis in tomato with the two alleles of *GAME9* may be suitable not only to regulate the core SGA biosynthetic pathway but also to regulate the upstream biosynthetic genes of the cholesterol pathway and possibly other genes that act upstream of this pathway.

**Fig. 4.**
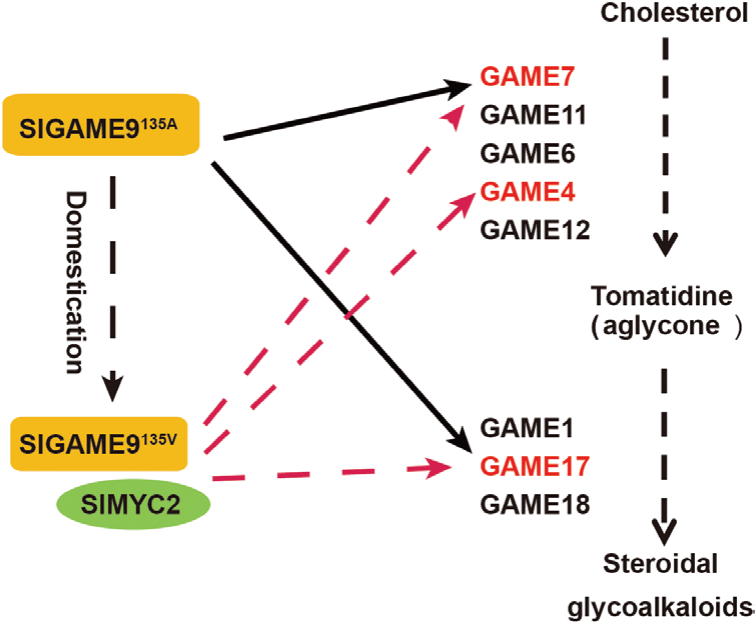
Influence of two haplotypes of GAME9 on the steroidal alkaloid pathway. GAME9 activates the synthesis of SGAs in tomato. *GAME9*^*135A*^ is mainly present in the PIM and CER populations and encodes a protein that can bind the *GAME7* and *GAME17* promoter. In contrast, *GAME9*^*135V*^ is mainly present in the BIG population and encodes a protein that shows weak binding to the *GAME17* promoter, and which might use an intermediate bHLH-like transcription factor, such as SlMYC2, to up-regulate SGA biosynthesis. (This figure is available in color at *JXB* online.)

In contrast to GAME9^135A^, which can closely bind the *GAME17* promoter, GAME9^135V^ showed weak binding activity with the *GAME17* promoter. However, how *GAME9*^135V^ ensures the flux of precursors in times of SGA production in the BIG population is still unclear. A previous study reported that GAME9^135V^ must bind another transcription factor, such as the jasmonate signaling component SlMYC2 (Solyc08g076930), to up-regulate *GAME4* and *GAME7* expression in a different scenario (Cárdenas *et al.*, 2016) (Fig. 4). Meanwhile, in *C. roseus* and *N. tabacum*, the AP2/ERFs *ORCA3* and *ERF189* are regulated by the basic helix–loop–helix (*bHLH*) transcription factors CrMYC2 and NtMYC2, respectively, which potentially function as activators of gene expression by binding G-boxes in the *ORCA3* and *ERF189* promoters (Shoji and Hashimoto, 2011; Zhang *et al.*, 2011). Previously, other studies reported that a jasmonate-inducible bHLH1 from *Nicotiana* enhanced the activity of ORCA1 (De Sutter *et al.*, 2005; De Boer *et al.*, 2011). Based on previous reports, we speculate that this cooperation may be more helpful and perhaps necessary to produce SGAs in the ‘low-SGA’ varieties where GAME9^135V^ has lower transactivation activity relative to the ‘high-SGA’ material, where bHLH-like transcription factors may be helpful but not necessary.

In general, a high concentration of glycoalkaloids and their byproducts adversely affects the nutritional value of tomato fruit and human health (Roddick, 1996). Thus, breeding tomato varieties that accumulate low levels of alkaloids and elucidating the underlying molecular mechanism that promotes the accumulation of SGAs to facilitate these breeding efforts are significantly important. In this study, we determined that natural variation in *GAME9* plays an important role in regulating SGA biosynthesis. Furthermore, in tomato, GAME9^135A^ can bind target gene promoters and regulate SGA biosynthesis. In contrast, GAME9^135V^ showed a weak binding activity with target gene promoters and may need to interact with an additional factor, such as SlMYC2, to enhance the binding activity with target gene promoters. Compared with *GAME9*^135A^, *GAME9*^135V^ appears to have relatively less influence on the production of SGAs. This molecular mechanism is entirely different from the previously reported mechanisms that lower the toxicity of SGAs and clearly explains the regulatory mechanism of toxicity. Moreover, this control strategy is probably one of the most crucial for up-regulating the flux of precursors in times of SGA production and for balancing essential phytosterol biosynthesis and the breeding of low alkaloid tomato varieties during their domestication. In tomato, the practical value of this work is that we identified the key allelic variant (SNP G/A, ch01:84029382) in *GAME9* that can be used to develop a CAPS maker that will be useful for breeding low alkaloid varieties of tomato with better nutritional value.

## Supplementary data

Supplementary data are available at *JXB* online.

Fig. S1. Manhattan plots for GWAS on six SGA contents.

Fig. S2. *In vivo* function of *GAME9*.

Fig. S3. SGA pathway-related gene expression at the mature green fruit stage in *GAME9*-RNAi lines.

Fig. S4. GAME9^135A^ binds the *GAME17* promoter and activates its expression.

Fig. S5. The relative expression of *GAME17* in red ripe tomato fruit.

Fig. S6. Alignment of GAME9 amino acid sequences.

Table S1. Primers used in this study.

Table S2. Genes within 122.7 kb of the SNP (ch01:84016748) most highly associated with fruit hydrotomatidine content

Table S3. The SNP and their P-value (associated with fruit hydrotomatidine content) in the 86 haploblocks.

Table S4. The GC-rich motifs found in the promoters of *GAME4*, *GAME7*, and *GAME17*.

eraa014_suppl_supplementary_figures_S1_S6_tables_S1_S4

eraa014_suppl_supplementary_table_S2

eraa014_suppl_supplementary_table_S3
