# Supplementary material for: An allelic variant of GAME9 determines its binding capacity with the GAME17 promoter in the regulation of steroidal glycoalkaloid biosynthesis in tomato
Source: J Exp Bot. 2020 Jan 14;71(9):2527–36. doi: 10.1093/jxb/eraa014 (PMC7210767; doi:10.1093/jxb/eraa014)
Supplement: eraa014_suppl_supplementary_figures_S1_S6_tables_S1_S4 [file eraa014_suppl_supplementary_figures_s1_s6_tables_s1_s4.pdf]

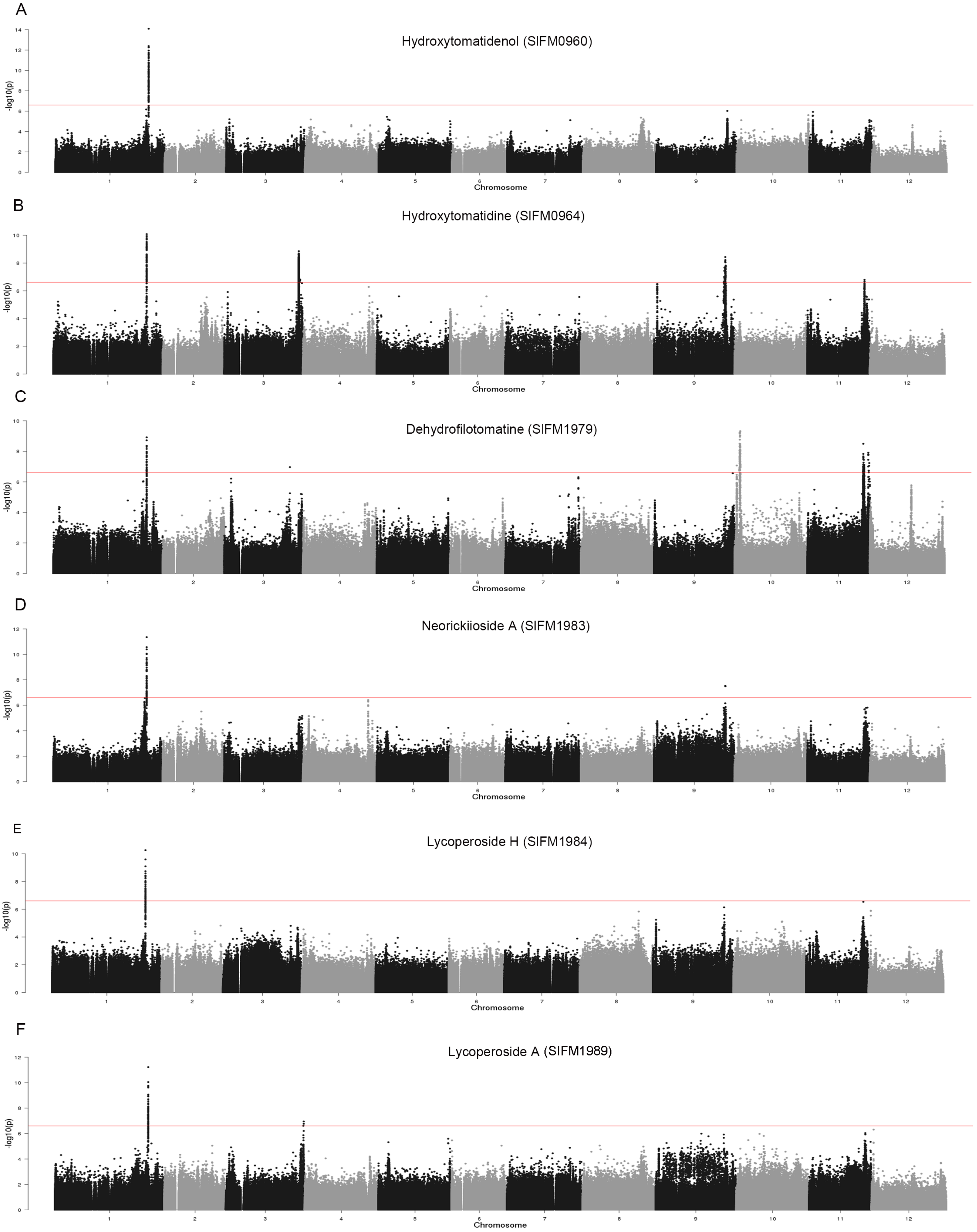

Fig. S1. Manhannttan plots for GWAS on six SGAs contents. Horizontal dashed line indicates a genome-wide suggestive threshold of 2.4×10^{-7}.

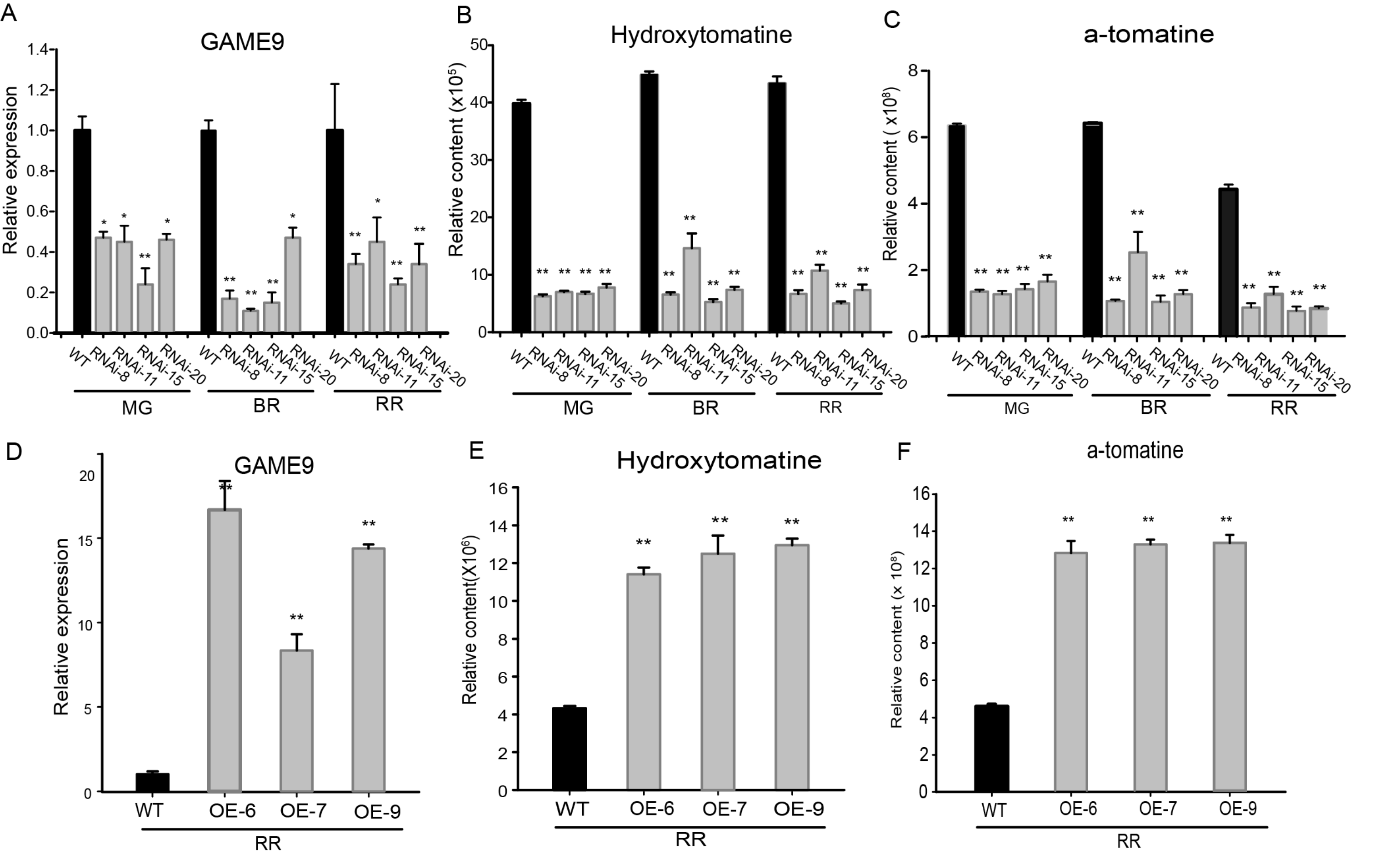

Fig. S2. In vivo function of *GAME9*. (A, D) *GAME9* gene expression (qRT-PCR) in the fruit tissue of *GAME9*-RNAi (silencing) and *GAME9*-OE (overexpression) lines. WT: wild-type (TS-286), independent *GAME9*-RNAi lines (8, 11, 15, 20), independent *GAME9*-OE lines (6, 7, 9). (B, E) Hydrotomatidine levels in the fruit of *GAME9*-RNAi lines (B) and *GAME9*-OE lines (E). (C, F) Levels of a-tomatine in the fruit of *GAME9*-RNAi lines (C) and *GAME9*-OE lines (F), quantified using LC-MS. MG, Mature Green; BR, breaker; RR, red ripe. Values represent means ± S.E. (n=3). Asterisks indicate statistically significant differences that were determined using a Student's t-test. *P-value < 0.05; **P-value <0.01.

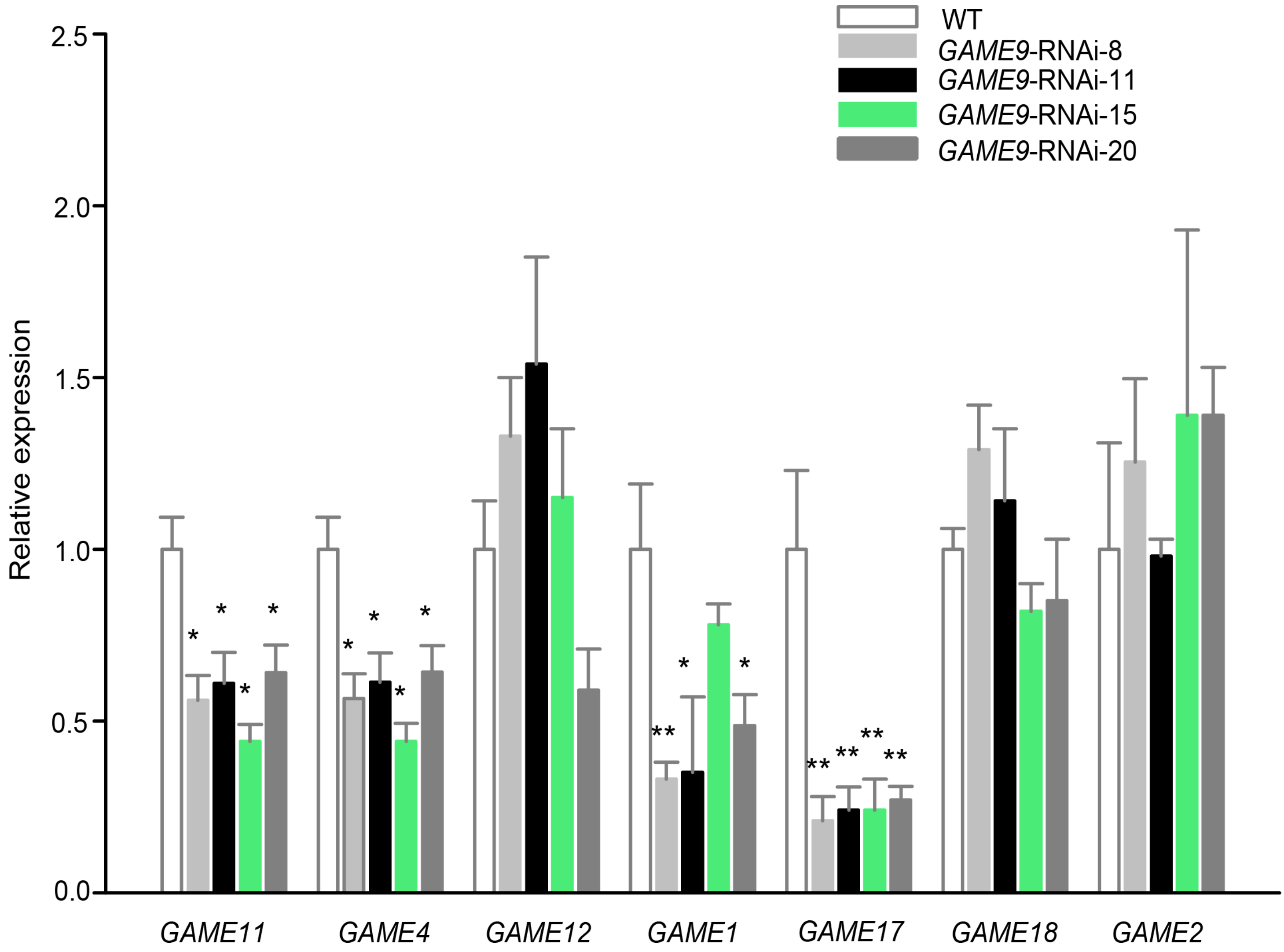

Fig. S3. SGA pathway related gene expression at the mature green fruit stage in *GAME9*-RNAi lines. Values represent means ± S.E. (n=3). Asterisks indicate statistically significant differences relative to wild type (TS-286) that were determined using a Student's t-test (*P-value < 0.05; **P-value <0.01).

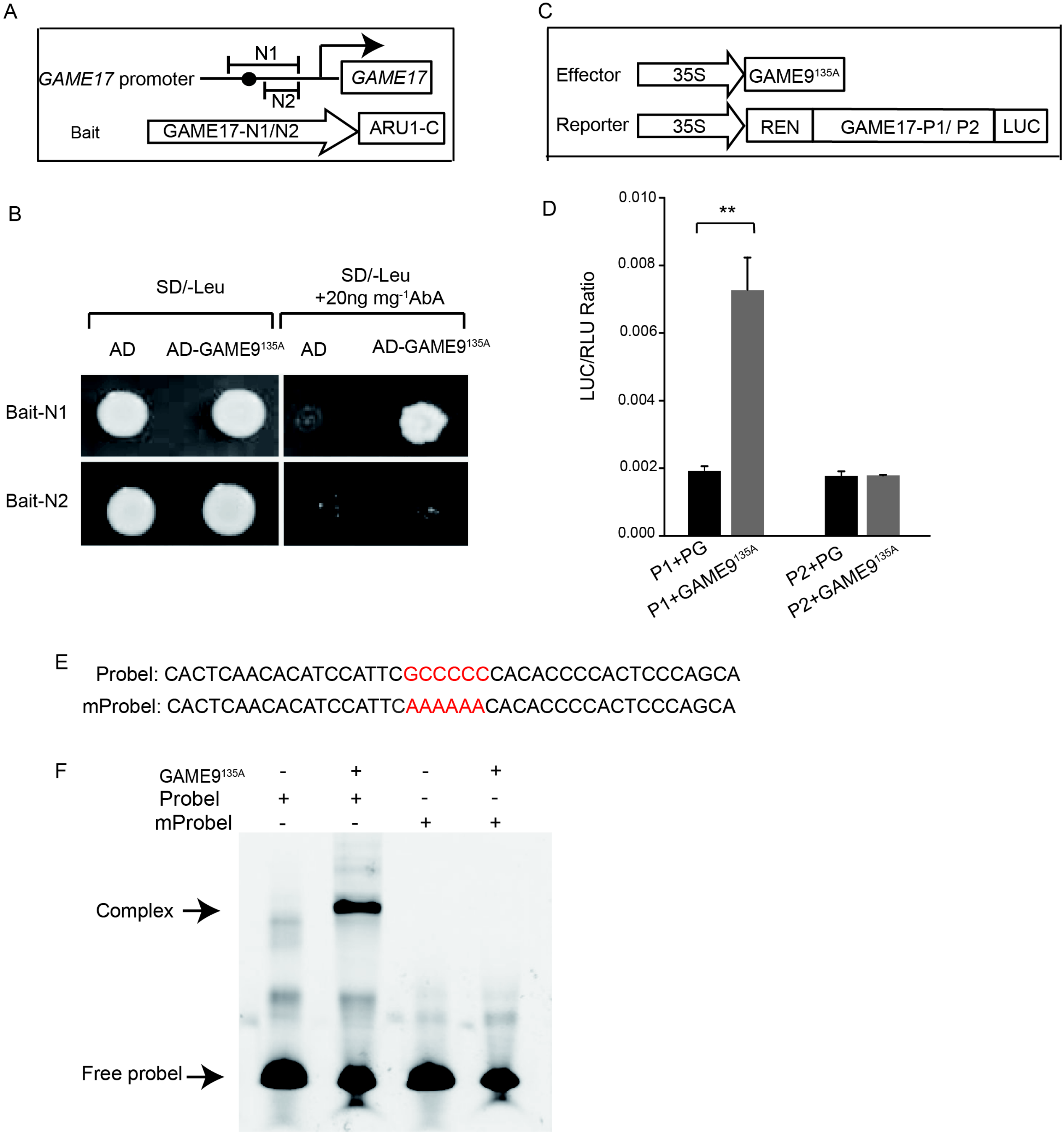

Fig. S4. GAME9^{135A} binds the *GAME17* promoter and activates its expression. (A) Schematic diagrams of the *GAME17* promoter and reporter constructs used for the Y1H assays. The circles indicate the cis-acting element, GC-rich element (-724 to -717 bp) within the *GAME17* promoter. N1 (-898 to -378 bp) and N2 (-666 to -378 bp) indicates the two promoter fragments of *GAME17* that were cloned into the bait plasmid pAbAi. (B) Binding of GAME9^{135A} to the *GAME17* promoter in yeast-one hybrid (Y1H) assays. The bait vector and the empty pGADT7 were co-transformed into Y1Gold as a negative control. All transformants were grown on a selective medium containing (left) or lacking (right) 20 ng mL^{-1} antibiotic (AbA).(C) Schematic diagrams of constructs used for the dual luciferase assay. Two promoter fragments from *GAME17*—P1 (-898 bp to the translational start codon) and P2 (-671bp to the translational start codon)—were cloned into pGreen II 0800-Luc to create reporter constructs. The full-length open reading frame of GAME9^{135A} was cloned into pGreen II 62-SK to create the effector construct. (D) Transcription activity assay in tobacco. This assay tested for interactions between GAME9^{135A} and the *GAME17* promoter. The P1 and P2 fragments and the pGreenII 62-SK empty vector (PG) were used as controls. LUC, firefly luciferase activity; RLU, Renilla luciferase activity. Bars indicate means ± S.E (n=5). Asterisks indicate statistically significant differences that were determined using a t test. ** $P < 0.01$. (E) Wild-type and mutant probes were used for EMSAs. The wild probe was synthesized based on the *GAME17* promoter sequence. The cis-element sequence was replaced with AAAAAA in the mutant probe. The hypothetical cis-element is indicated with red letters. (F) In vitro binding of GAME9^{135A} to the promoter of *GAME17*. -, absence; +, presence. The protein-DNA complex and free probe are indicated.

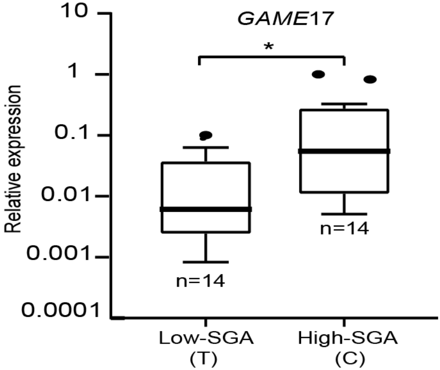

Fig. S5. The relative expression of *GAME*17 in red ripe tomato fruit. n refer to the number of appropriate genotype of tomato accessions were used in this study, the middle line of the box indicates the median, the box indicates the range of the 25th to 75th percentiles of the total date, the whiskers indicatethe interquartile range and the outer dots are outliers. Asterisks indicate statistically significant differences that were determined using a t test. *P < 0.05.

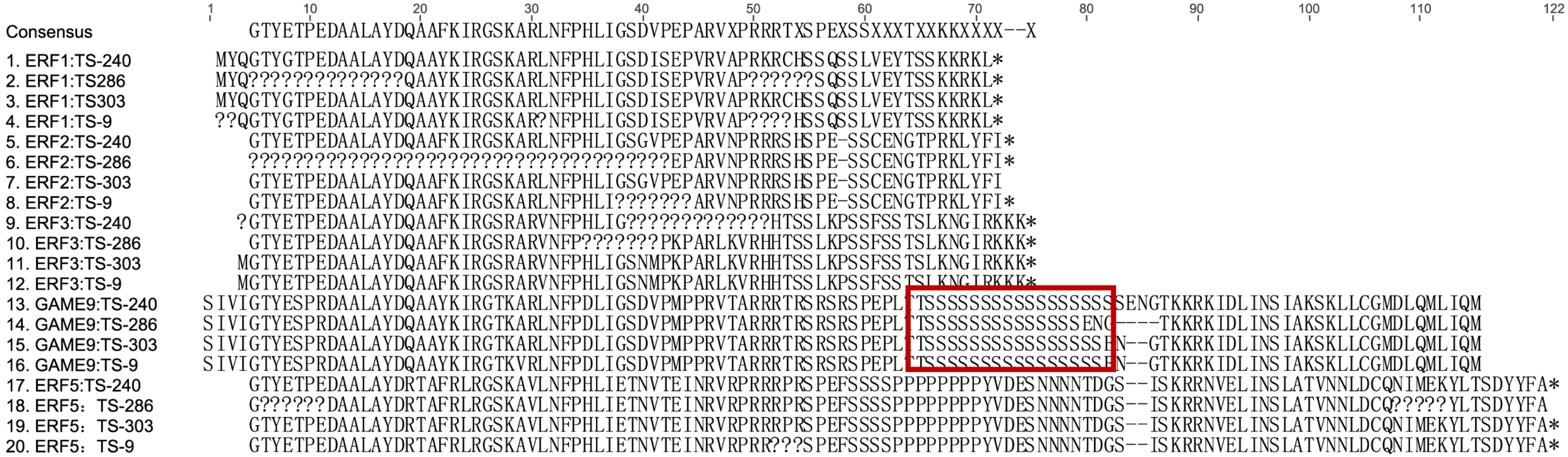

Fig. S6. Alignment of GAME9 amino acid sequences. Amino acid sequences of GAME9-like proteins amplified from resequencing data of different tomatoes (TS-240, TS-286, TS-303 and TS-9) were aligned with the Geneious. The serine-rich C-terminal domains are highlighted with a red box. ERF1, ERF2, ERF3 and ERF5 are GAME9-like genes in this tandem duplication.

Table S1. Primers used in this study.

name	sequence	note
GAME17-FW1	AGCTTGAATTCGAGCTCGGTACC CAAGACGCAACATGAATTTAACTCC	Y1H(N1)
GAME17-RV1	ACATACAGAGCACATGCCTCGAG CGACACATGAAATTTTGAAATTCAA	Y1H(N1)
GAME17-FW2	AGCTTGAATTCGAGCTCGGTACC GTGAAAAGGTAGTAAATTCTTCTTC	Y1H(N2)
GAME17-RV2	ACATACAGAGCACATGCCTCGAG CGACACATGAAATTTTGAAATTCAA	Y1H(N2)
GAME9-Fw	CATTAACAACTTCGTCCTCGTCATC	qRT-PCR
GAME9-Rv	ATCCCACAAAGTAATTTGGATTTTG	qRT-PCR
GAME18-Fw	TAGCTTATGGCCACATCACACCTTT	qRT-PCR
GAME18-Rv	TTGCCTTGATGAAGCTAAGATTGAT	qRT-PCR
GAME1-Fw	AAAACCTCCATGTTCTGTTCCTTCC	qRT-PCR
GAME1-Rv	GAGGGGTAGTGAGGATTGTGGCTTT	qRT-PCR
GAME17-Fw	TCTAGTCGCGGAAGTGCTAAAAATA	qRT-PCR
GAME17-Rv	TATCACCTTCTTCCGATGCCATTAA	qRT-PCR
GAME2-Fw	ATGGCGATGGAAGAGAATGAACAAA	qRT-PCR
GAME2-Rv	GCCATGGAAGGCCAAGAGTCTAGCA	qRT-PCR
GAME4-Fw	TAGCCTTGTTCTTCATAGCTTTAAT	qRT-PCR
GAME4-Rv	TGTTTGAACAAAATTTGATTGCATA	qRT-PCR
GAME12-Fw	ATTTCATTTCTTGCTTTATCTTTCT	qRT-PCR
GAME12-Rv	AACATATCATGTCCCATAAATCCAT	qRT-PCR
GAME6-Fw	TAGCTTTGGCCATATTTTTTCCTTT	qRT-PCR
GAME6-Rv	CATAGATTCCTTGATGACGCAGTTC	qRT-PCR
GAME11-Fw	GATCTAATGGATGAAGCAATGAAAG	qRT-PCR
GAME11-Rv	CCCCTATTGGTATTATTAGCTGCAT	qRT-PCR
GAME7-Fw	TCCTCGTTTTTTACACACAAATTCA	qRT-PCR
GAME7-Rv	CAATTGCTATGGCAGAACAACACAC	qRT-PCR
GAME9-OE-Fw	CCTCGAGCACATATTATTCATCCAAAACAAGA	Cloning *GAME9* for OE
GAME9-OE-Rv	GGGTACCACACAATGACCTTGATGAGTCTATT	Cloning *GAME9* for OE
GAME9-RNAi-Fw	GGGGACAAGTTTGTACAAAAAAGCAGGCT TTTCCCCAATGAGTTTAGATGTTAC	Cloning *GAME9* for RNAi
GAME9-RNAi-Rv	GGGGACCACTTTGTACAAGAAAGCTGGGT TTACTCTAGGTGGCATAGGTACGTC	Cloning *GAME9* for RNAi
GAME9-62SK-Fw	GCCGCTCTAGAACTAGTGGATCC ACATTCATCACATATTATTCATCCA	Used for transient expression assays
GAME9-62SK-Rv	TTGGTACCGGGCCCCCCCTCGAG GTGAAAGAAGTGCATTTACACAATG	Used for transient expression assays

Pro1*GAME17*-Fw	CACTATAGGGCGAATTGGGTACC<br>CAAGACGCAACATGAATTTAACTCC	Used for transient expression assays
Pro2*GAME17*-Fw	CACTATAGGGCGAATTGGGTACC<br>CGCACGTGAAAAGGTAGTAAATTCT	Used for transient expression assays
Pro*GAME17*-Rv	TATGTTTTTGGCGTCTTCCATGG<br>GTGTGAAGATGTTGTGGAGTATAAC	Used for transient expression assays
GAME9 -Fw	GTACCAGATTACGCTCATATG<br>ACATTCATCACATATTATTCATCCA	Y1H
GAME9 -Rv	ATGCCCACCCGGGTGGAATTC<br>GTGAAAGAAGTGCATTTACACAATG	Y1H
GAME17-FW	CACTCAACACATCCATTCGCCCCCCAC<br>ACCCCACTCCCAGCA	EMSA
GAME17-RV	TGCTGGGAGTGGGGTGTGGGGGGCGA<br>ATGGATGTGTTGAGTG	EMSA
GAME17-FW (mutated element)	CACTCAACACATCCATTCAAAAAACAC<br>ACCCCACTCCCAGCA	EMSA (negative control)
GAME17-RV (mutated element)	TGCTGGGAGTGGGGTGTGTTTTTTGAA<br>TGGATGTGTTGAGTG	EMSA (negative control)
Pro*GAME7*-FW	CACTATAGGGCGAATTGGGTACC<br>GAGGCAAAGATGACTCTCTGTATGA	Used for transient expression assays
Pro*GAME7*-RV	TATGTTTTTGGCGTCTTCCATGG<br>TTTGTTTGAGTTGAATTTGTGTGTA	Used for transient expression assays
Pro*GAME4*-FW	CACTATAGGGCGAATTGGGTACC<br>GAATCTGATACTACTAATATTAAAT	Used for transient expression assays
Pro*GAME4*-RV	TATGTTTTTGGCGTCTTCCATGG<br>TGTTCTCTCTCTCTATTTTTTTTC	Used for transient expression assays
Pro*GAME18*-FW	CACTATAGGGCGAATTGGGTACC<br>TGATTTCTAGGTATTAGCAATGCAA	Used for transient expression assays
Pro*GAME18*-RV	TATGTTTTTGGCGTCTTCCATGG<br>CACTTGTGTCTCTGTATTTTTTTCT	Used for transient expression assays
Pro*GAME1*-FW	CACTATAGGGCGAATTGGGTACC<br>GGCTAGATCCCTGAGATGTTAATTA	Used for transient expression assays
Pro*GAME1*-RV	TATGTTTTTGGCGTCTTCCATGG<br>TTTTACTACCCCAATACAAAGAACA	Used for transient expression assays
Pro*GAME12*-FW	CACTATAGGGCGAATTGGGTACC<br>CACCGATCTTTGGAAAAAACTCATT	Used for transient expression assays
Pro*GAME12*-RV	TATGTTTTTGGCGTCTTCCATGG<br>TCCTTGAAGCTTTTTTGAAAAATTA	Used for transient expression assays
Pro*GAME6*-FW	CACTATAGGGCGAATTGGGTACC<br>TTTTTGTGCGTGTTTATGAGCTTTC	Used for transient expression assays
Pro*GAME6*-Rv	TATGTTTTTGGCGTCTTCCATGG<br>GTGTTTGTTTCTTCTCTCCCTTTGC	Used for transient expression assays

Pro*GAME11*-FW	CACTATAGGGCGAATTGGGTACC GGACCCAACCCGATTTATGTTCTAC	Used for transient expression assays
Pro*GAME11*-RV	TATGTTTTTGGCGTCTTCCATGG TGACAATCAATCTAAACCAAAACCA	Used for transient expression assays
Pro*GAME2*-FW	CACTATAGGGCGAATTGGGTACC TTTGAACCCTTACCCTAATTCTCTG	Used for transient expression assays
Pro*GAME2*-RV	TATGTTTTTGGCGTCTTCCATGG TTTCACTACATTTGAACTTGTTCCT	Used for transient expression assays
Pro*GAME17* -Fw	TGCATCCAACGCGTTGGGAGCTC CAAGACGCAACATGAATTTAACTCC	Used for GUS activity assays
Pro*GAME17*-RV	GCCTTCGCCATTCTAGACTCGAG GTGTGAAGATGTTGTGGAGTATAAC	Used for GUS activity assays
GUS-FW	AAGTGTCGGTCGTGGATGAG	Used for GUS expression assays
GUS-RV	AGTCCGTCGTTCACCAGTTC	Used for GUS expression assays
Q-actin-FW-1	GTCCTCTTCCAGCCATCCAT	qRT-PCR
Q-actin-RV-1	ACCACTGAGCACAATGTTACCG	qRT-PCR

Table S4. The GC-rich motifs found in the promoters of *GAME4*, *GAME7,* and *GAME17*. The presence of GC-rich motifs (yellow) used in the transactivation assays and the coding sequence (red) are highlighted.

Solyc12g006460 **Cytochrome P450 (*GAME4*)**

>SL2.50ch12:939904..934011

GAATCTGATACTACTAATATTAAATTTAAAGCCAACAACCCCGCAAAATAAATCTTCA
CGTGACCAATGCATCAAGTGGAATTTATTAAAATTAAATACGACAAGATATGTAGAA
AATTATTGTTACAAAAATTAGAAAGAAACAAAGTCCATCTTTGAATTCTCTCACATAA
AATATTGGGACCCAACGTTAGAAACATTATTGTTATGGCAAAAGAAAAAATCGGTTA
TCATCGACGTATCTGATCCAGTAGATAAGATTATTTCTCTCGTTAATCGTAGGTTTTGA
ATTTGATTTTTTAGTATTAAAAAAAATTTTTGGTAAGAAATGTCTTTCCCTATCCTTCT
AATGGAATCCTATGTAGCGCGAATTCGAAATAGTCTAATTTCAATTTAATTATTAAAC
ACAAAATGAAAATAAAAACAAAAATCCTCATTTTACTAATAATCATAATAATGATGT
AGAAATTATTAGCTAATCCCAAAATATTAATCATAAATAAAATTTAACTAATAATCAA
AATAAATATTATATATAAACCAACAATCAAAACAAAAACTCATAGTATAAGACAGTA
CAACAAGTGTAAAGGTTGTGTAAAAGCTTTTAGGTTTCTAGTCAAATAACTATGATTT
TTCTTCAAAAATATCTTAAAGAATTATATATATAATCAATATATGATTTTTCATTTTTT
TTGGTTTATTATTATTTTAGTTTTTCCCTTTCTTTTAATTTTTTCCATACCTTTTGTCCGT
AATCATGGACCATATCATTATTTGTTAACTGAAATGACAAAAATAAACCAAAATTTAA
CAGCTAACATAAATAAGTTTTTCTCAAAATTCAACAACACATTTAAACCTTTTCATTT
AAAAAAAAACAATTTCTTTAATAATTTTACAAAATCGAAAATATATTTAGTTTATCAA
TAATGAAACCTTTTAACGAAAGTTGGGGGGCTAAATTATATTCACGTGATTGTATTTT
TTTTTTTAATTGCACTTCTATTAAGGAATAAAATTATAAAGATTTAACTATCTTTGAAA
GTAAAAAGGTTAGTATTGTCTTTTCACATATTTGGTCTCATCATTATTGTTGCCATATT
TTTTTAGATAAATAAATGTAGCCATTTACTACCTCCAATCAAATTCACAAATCCATCC
AAATTATTCAAAGAAAAAAAAATAGAGAGAGAGAACAATGGATTTCTACAATTTAGC
CTTGTTCTTCATAGCTTTAATACTTGGAATTTTCACATTTTATGCCATATTAATGAGAA
TAAATGGTTGGTATTATGCAATCAAATTTTGTTCAAACAAATATAACATCCCAAATGG
TTATATGGGTTTGCCATATTTTGGTAACACACTTTCTTACTTCAAAGCTTCAATGTGTG
GTGATCCAAAATCATTCATTGATTTCTTTGCTACTAGGTTTGGAGAAGGAGGAATGTA
TAGGGCATACATATTTGGGAAGCCAACAATTATGGTGACAAAGCCAGAAATAATTAG
AAAAGTTTTGATGGATGAAGAGTATCTTGAAAGAGGTTTGCCTAATTATATGAAAAA
ATTAATTGGATTAACAACTTCGATAGAAGAAGACAAATATTTTCGTAGATTAACAGC
ACCAGTAAAAAGTCATGGATTATTATCTGATTATTTCGATTATATCGATAAAACTGTG
AGTTCTACATTAGAGAAATACGCTACTACGGAAGAACCTGTTGAGTTTCTTCATAAAA
TGCACAAGCTTACGTTTGAGGTGTTTATGAGACTTTTAATTGGTGATGAAGTTAATCA
AGAATTATTTGATGAAATGTTTGAGGAGATTACTGCTGTAATTAGTGGTGTTCACAAT
TTGCCAATTAATCTCCCAGGATTTGCTTATCATAAGGGACTCAAGGCTCGAAAAGTAC
TAGGAGAGGTATTTAAAAAATTAATTGATGAAAGAAGAGAAGCCATGAAGGATGGA
AAATCAATGCCAAAGGCAAACATAATTGATATGTTGTTATCAAACAACAATCAAGAT
TATGAAGCAAACATGTTGAGTGACAAGAAGATCATTGAAATCCTAGTTTTGTTTTCAT
TTGCTGGTTTTGAACCTGTTGCTCTTATGTCTGTCAAGGCAATTTTCCACTTACAAAAA

CATCCACATTTCTTGGAAAAAGCCAAAGAGGAACAAGAGGAAATAGTAAAGAGAAG
AGCATCTTCAAATGCTGGACTTAGTTTTGATGAAATTAGACAAATGACATTTGTTAGT
AAGATAATTAATGAAACGTTACGTATAGCTACTGATCAGTCGGTATTCCTTAGAGACA
CAAGTACTACTTTTAACATAAATGGGTACACCATACCCAAAGGGTGGAAGTTTTTTGC
AGTTGTATGGAATATTCATATGAATCCTGATGTTTATGTTCAACCTAAGGAATTTAAT
CCTTCGAGATGGGATGATATTGAAACTAAGCCAGGCATTTTTCTACCTTTTTCAATGG
GCCCCAAATCATGCCCAGGATCCAATTTGGCCAAGCTTCAAATTTCAGTAATTCTTCA
TTATTATCTTCTTCACTACAGGGTTGAGCAAATTAATCCAGAGGCTAGATGTTATCCT
CCTGAAAATTGTCTTGTGAAATTCAAGAAGCTATCGATCTCTAGTAATGGTAATTAA

Solyc07g062520 **CYP72(*GAME7*)**

>SL2.5ch07:65219309..65223512

GAGGCAAAGATGACTCTCTGTATGATCCTACAAAGCTTCTCTTTTGAATTGTCTCCATCC
TACACACATGCGCCTCAACCCCTACTTACCATTCATCCTCAGTATGGTGCTCCTCTTATT
TTCCACAAACTATAATTTCATATTACGTGAGGTATCTTTCACTTGTACGACTATACTGGAT
ATGTTATTGTTCAGACAAACTCAATAACTCTTGTTTAAACCTTTTATCTGTACCAGAGAA
TAAATAACATGTTGCAAATTATATACAAATCATCTCAAGAACCAATGAGAAGCTTATTGT
TATGACAAATTTAGATATATTCTACAAACACATTAAGCTAAAGAATTTGTGGAAAAAATA
ACTGTTTTTTTTTTTGGTCACAATAAAAACATGTGACATGCTAGTAAGCAGTAAGCACA
GCCGGCCTTTTTGGCAATGAAACCACTACATACACTTGTACACTAATTCTTTTTTCATTT
TTTAAACTAAAATTATGAATGTCAGAATACGAAATAACAAGTAAAAACAAACGAAAAT
GATTAAAAATATAAGAGAAAATCAGAGCCACTCATTGGTGTCTTTGCTCATCATTGTTG
ACGACGTCGTTTGTTAGTTCCCAGAGACATAGTATATATAAATTAATACTCCTCGTTTTTT
ACACACAAATTCAACTCAAACAAAATGGACGAAATTCAAATATTAGTAAGAGTGTGTT
GTTCTGCCATAGCAATTGCTCTGTTTGTGTGTCTGTGGAAAGTACTAAATTGGGTTTGG
CTCAATCCGAAGAAGTTGGAGAATTTGTTGAGGAAACAAGGGCTAAATGGGAACTCTT
ACAAAATATTGTATGGGGATTTGAATGATTTTATTGGAATGATTAGGGAAGCTAGTTCAA
AACCTATGAATTTGTCTGATGATATAGCTCCAAGATTGGTGCCTTTCTTTCTTGAGACCA
CCAAGAAATATGGGAAAAAATGTTTTATATGGTTGGGTCCAAAACCACAAGTAATAATC
ATGGACCCTGAGCTTATTAAGGAAGTACTCTCGAAAACCTATTTGTATCAAAAGCCCGG
TGGAAATCCATTTGCAGCATTATTGGTACAAGGACTAGCAACCTATGAAGAAGATAAAT
GGGCCAAACATAGAAAAATTATCAATCCCGCTTTCCATCTAGAGAAGCTTAAGCATATG
CTCCCAGCTTTTCACTTGAGCTGTACTGAAATGCTGAGCAAATGGGAAGATGCTGTTCC
ACTTGGCAGCTCTCGTGAGATAGATGTATGGCCTCACCTTCAGCAATTAACTTGTGATG
TAATCTCTCGGACAGCTTTTGGAAGTAGTTATGAAGAAGGTAGAAAGATTTTTGAACTT
CAAACGGAACAAGCTCAGAATTTTATCGACGCTGTACGTGAAGTTTATATCCCAGGGCG
GAGATTTTTGCCAACAAAGAGGAACAGAAGAATGAAGGAAATAAAAAACGAAGTTCG
GACCTCAGTTAAAGGTATTATCGATAAAAGAATGAAGGCAATGAAAGCAGGGAATGCC
GATAATAATGAGGATCTGTTAGGCATATTGCTTGAATCAAATTTCAAAGAAATTGAACA
GCACGGTAACAAGGATTTTGGAATGAGCATCGAAGAAGTCATCGAAGAATGCAAGTTA
TTCTATTTTGCTGGCCAAGAAACTACATCAGTCTTACTCGTGTGGACTCTGGTGTTGTT
GAGTCGGTATCAAGATTGGCAGACACAGGCCAGAGAAGAAGTCTTGCAAGTGTTTGA
GAGTCGGAAACCAGATTTTGATGGATTAAATCATCTAAAAGTTGTAACAATGATCTTGT
ACGAGTCATTACGGCTATATCCCCCATTGATAACACTTAATCGACAGGTTAACGAAGAC

ATAGTATTAGGAGAATTGTGTCTACCAGCTGGTGTACTAGTCTCATTGCCAATGATCTTA
TTGCATCATGATAAAGATATATGGGGTGAAGATGCAAACGAGTTCAATCCAGAGAGATT
TAGAGAAGGAATATCAAGTGCAACAAAAGGTAAAGTTACATATTTTCCATTTTCATGGG
GTCCAAGAATATGCATTGGACAAAATTTTGCCATGTTAGAAGCAAAGATGGCTTTGTGT
ATGATCCTACAAAGCTTCTCTTTTGAATTGTCTCCATCTTACACACATGCTCCTAAATCC
TTAGTAACCATGCAGCCTCAGTATGGTGCTCCACTTATTTTGCACAAACTTTAG

Solyc07g043480 **UDP-glucose glucosyltransferase (*GAME17*)**

>SL2.50 ch07: 57268672..57270940

CAAGACGCAACATGAATTTAACTCCTTTTCATTATTTATTCTTTTCATTTTTCAAGTAT
ATATTTGGATGAAAATCATATCTTATAATTAAAAATTTATTTATTATTTTAAATCATCA
CACATGACTTCTATTTAATTAATAAAAAGATATGTATTCACTCAACACATCCATTCGC
CCCCCACACCCCACTCCCAGCACCTATTTAACATACAGTACACCCATTATGCGCACGT
GAAAAGGTAGTAAATTCTTCTTCTTCTTCTATATAATAAAAAATAAAAACTAAATTTT
TAAAATTTTATTTGTGTCATATAAATTGAAACAGTGAAAATAATGTATATTATTAGTG
ACAATAATTAATGATAAACGACATTTGATTGAATTTTAAATTTCA*CACGTG*ATACATA
GATTGAGATTATTATTTGACAATTAAAATTTAAATTAACAAATTACAATAACTGACAA
CTAAATTTTTATTTTTATTAAAAGTTTGATTGAATTTCAAAATTTCATGTGTCGAATAA
ATTGAGATAGAAATAGTTATATATATATATGTATATCAATTGAAAACTATTTTAGAAG
TATTATAAATCATAATGATTAATATATAATTTAAAGTAGTTAAAATTTAACATGATTC
AAAATACATAAATTAATGGACTTATTTATGATATTTTCTTCTCAACACATTGAAGTAT
ATATTTGGAAAAATGATGAATCTAAATTTTAGGCTGTCTGTTTAAATTATTATTTTCAT
ATGCAAACATTAAAAATACGGTTGTTGGTATTCGGTAGTTGTTACTTCATATCTATAT
ATCCTTGTTTAGAGTAACACTCCAATAAGTCTTCAAAAAAAAAAAAAAAATACTAATA
GTTATACTCCACAACATCTTCACACATGGATAACAAAGATGATGTAGCTGTTGTGATA
GCTCCATTACATCTTCAAAGCCATCTCCGACAACTTCTCCATTTTGCATGTAGAATTTC
CTCCTATGGTCTACCGGTCTACTATCTCGGCTTAGCCGCCTCTAACCGTGAAGCTAGG
CAACACTCGACTACCTTAAATCCTTGTGACATAGAGAAAATCCACTTCCATGACCTCC
AAATTCCTAATGAAGATCCTCCTCCAGATATTCCCATGCATATTTGGCATGCTTCTATG
CGAACACGTGAACCCATTGCTTCTTTCCTTGGAGATATCTCCTCAAAGGCAAGACGAA
TTGTTGTTGTTCATGATCTTTTAATGTCCTATAATGTTCAGGATATTTCTTCATATCCCA
ATGGAGAATCCTATGTATTTCATTGCCTACCTATTTTCGATATGTATTGTAGTCACTAT
GCCCCAGCTGCAGGATTCCCTATTCCACTTGAAGAAGCGCTTCTTAAAAGGCTACCTT
CCAATGATGGATGTTACAACCCTGAGGATGTGGAGCACCATGCTAAGTATCTAAATC
ATTGCATGGGCAAGAATGCTGGTGATATCTTTAACACAAGTCAAGTAATTGATGGTAC
TGCTATTGTTGATTCCATGGCAAATCTTGCCACCATGCAAAACAAAAAGTTATGGGCA
TTAGGACCAATTCTCCTTACACAAGATCATGATAAGGTCCAAACCAAACACTTCTGTT
TGGATTGGCTTAACAAACAGCCTCCTAAATCAGTTATTTATGTGTCTTTTGGAACATCT
ACTTCATTTTCAGCTGAACAAATCAAGGAGCTAGCAATTGGTTTAGAGCTAAGCAAA
CAAAAGTTCATATGGGTACTTAGAAATGCTGATGTAGGAGATCCTGTTAACAACAAA
TGCGAAGAGAATAGATCAGGAACACTTGAGTTGCCAGAAGGATTTGAAGAAAGGGT
AAAAGGGGTTGGCTTAGTGGTGAGAGAATGGGCACCTCAACAAGAAATCTTCGCTCA
TCCCTCTACTGGTGGCTTCATGAGTCACTGTGGATGGACTTCGTGTTTAGAAAGCATT
ATTGAAGGTGTGCCAATAGCCGCTTGGCCTATCCAATTTGATCAACCAAAAAATGCTT

TTCTAGTCGCGGAAGTGCTAAAAATAGGGCTCCATGTTAGGGAGTGGGAGCAGCGGA
ATGAGTTAGTGACTGCTTCCACCGTTCAAAATATAGTTTGCAAATTAATGGCATCGGA
AGAAGGTGATATGATTAGGAAAAGAGCACAAGAACTAAGGACGGCTGTAAAGCGGT
CCACGGAGGAAGGTGGTGTTTCTCGAATGGAGTTGGATTCTTTCATCGCGCATATCAC
AAGATAG
